# Ovarian Tumors

**Published:** 1862-02

**Authors:** Wm. H. Byford

**Affiliations:** Professor of Obstetrics and Diseases of Women and Children, in the Medical Department, Lind University, Chicago, Illinois


THE
### CHICAGO MEDICAL EXAMINER

N. S. DAVIS, M. D., Editor.---FRANK W. REILLY, M. D., Ass't Editor.

VOL. IV.] FEBRUARY, 1862. [NO. II

### Original Contributions.

ARTICLE V.

### OVARIAN TUMORS.

By WM. H. BYFORD, A. M., M. D.,

PROFESSOR OF OBSTETRICS AND DISEASES OF WOMEN AND CHILDREN, IN THE MEDICAL DEPARTMENT, LIND UNIVERSITY, CHICAGO, ILLINOIS.

### III.—Causes and Prognosis of Ovarian Dropsy.

It is extremely doubtful whether there is anything in the general condition of the patients that predisposes to the development of ovarian tumors. There is quite a disposition, however, with a certain class of authors, as will be apparent to any careful reader, to trace most chronic enlargements to scrofulous taint in the system; and these gentlemen express the belief that scrofula predisposes to ovarian disease,—in what way we are not informed. I think that no general conditions, but those resulting from age, have anything to do with their predisposition. The term of life during which menstrual influences are exerted upon the general organization, is very much more frequently the time when it is most active. So that we may very safely conclude that in the function of menstruation we have a predisposing cause of ovarian disease. It is true that ovarian tumors have been found in the ovaria of infants and fœti, and originating in very aged females; but this probably is as rare an exception to the general rule, that they occur during menstrual life, as the occurrence of menstruation in infancy and old age is to the general rule that this takes place at the usual time. And probably, too, a careful examination of the anatomy of these exceptional cases, will prove them to be as exceptional in nature to the ordinary ovarian tumor, as the time in which they occur would seem to indicate. Some circumstances connected with menstrual life, would seem also to increase the predisposition. Sixty-one per cent., according to WEST, of the patients are married, while only twenty-nine had never been married. After making allowances for the greater proportion of ladies at twenty-five who are married, I think we may fairly infer that marriage adds somewhat to the chances of the occurrence of ovarian dropsy.

That patients who are the subjects of this disease should be less likely to have children, than those in whom ovulation is more perfect and complete, will not, I think, justify us in setting down sterility as the cause of it in any way, but it is more probably connected as an effect. During menstrual life, the most obnoxious time is between the ages of twenty-five and forty, the time when the sexual functions are exercised with more activity than any other. So that it would be right to say that menstruation and marriage both predispose to ovarian disease.

Unhealthy menstruation seems to be more commonly coincident with it than healthy. Abortions and premature labor are so, likewise.

We should attach sufficient importance to the fact that it occurs in unmarried persons as often as twenty-nine per cent. This induces Dr. WEST to remark, that "it occurs in the unmarried oftener than any other organic disease of the sexual organs."

The exciting or proximate causes are such as excite the ovaria and induce abortive efforts at ovulation ; what does so, we are not able to say with certainty, but we can say what might and consequently does produce this effect.

Inflammation of a low grade and somewhat chronic duration, might cause induration or thickening of the indusium, so that it would not yield to the upheaving pressure of the ovisac, so as to burst or become absorbed and permit dehiscence, or the peripheral portion of the proper substance of the ovary might be condensed by it, and thus rendered unfit for a covering for the tumor.

The probabilities, I think, are quite in favor of this mode of initiating these morbid growths, or merging a healthy into an unhealthy accumulation. When once thus commenced, the stimulus of increased incretion of fluid would carry on a kind of hypertrophy in the involucra that would permit of a further enlargement. Now the local circumstances that are regarded as the causes of the disease, would favor the occurrence of inflammation, and are very frequently attended with some of the symptoms of it. The local condition of the ovary and uterus during each menstrual period, is often attended with pain in the ovarian region of just such a character as we would expect to indicate inflammation. This ovarian pain is present in other excited conditions of the sexual organs also, thus showing that they are very frequently the focus of painful vascular turgescence, if not inflammation. How easy it is to conceive the possibility, nay, the probability of nutritional vitiation to an extent sufficient to prevent dehiscence of the ovum after it is matured in the ovisac. If this state of things may take place in consequence of menstrual, or congestions from other transient or mild causes, how much more likely is it to be induced by the inflammations which succeed to abortions or parturitions. While inflammation is probably the cause of the beginning of the development of ovarian tumors, it does not seem necessary to their continued development, as the accumulation of fluid in a shut cavity with a secreting internal surface, is a matter of course, and the limit of its amount, for the most part, does not depend upon any thing but the capacity of the involucra to grow, which they usually do until interrupted by external circumstances.

Although inflammation may, in most cases, be the cause of the toughness of the covering to the ovary, which prevents the escape of the ovum, this condition may result from some other local circumstances. Congenital formation may be such as to give rise to this difficulty,—possibly, may allow develop ment of the ovisac; may allow the involucra to increase as fast as the demand for more room becomes necessary.

Our knowledge with regard to the prognosis, is unfortunately too definite. There is no need of much conjecture with reference to this matter; the termination is too frequently demonstrated. In arriving at prognosis, with reference to any disease, we ought to consider whether its ordinary course is, after a time, to a termination in health, as is the case with many diseases, or, there being no such favorable tendency, what are the probabilities of a cure. Unfortunately, there is no tendency to spontaneous recovery, worth taking into consideration; in ovarian dropsy, probably not two per cent., but would, after a longer or shorter time, terminate in the death of the patient. While this is the case, it does not properly represent the value of a life, threatened by this affection. Some patients live a great many years in comparative comfort; but, by a large odds, the case is generally very different,—only a few years being sufficient to finish th course in a downward direction. The average duration of life is probably about five years from the time it is first perceived. So much difference of opinion exists among observers, and so unsatisfactory are the statistics, that the average number of recoveries under treatment, is quite doubtful. The mortality taken altogether, I should say, is not far from fifty per cent.,—a fearful fatality, and the average duration of life under treatment, and in spontaneous cures, will not exceed very much, if at all, seven years. Yet, in *selected* cases, probably two-thirds are so that the patients will enjoy perfect health. Much of the mortality, in my estimation, depends upon the injudicious selection of cases for the different modes of treatment, which have of late years, proved successful. However discouraging the prognosis, in general, may appear in this kind of examination, we are not justified in exterminating the hope of our patient, by applying it to her case without particular investigation into the peculiarities of it. In fact, individual prognosis is too often left out of view, and general results are applied to individual cases. In certain instances, either too favorable or unfavorable, an opinion is yet to be expressed, to the undue elation of our patient, or the perfect annihilation of all her hopes. It cannot be the case, that all instances of the most unfavorable diseases, are to be unconditionally consigned to death; nor that any one of the more favorable classes of disease is to terminate in health, without question.

We should, therefore, carefully examine every individual case, with reference to its own peculiarities, its nature, and the character and condition of the patient. Is the disease simple, or compound of cyst and solid, polycystic, or monocystic? The monocystic is very much more favorable for treatment, and terminates in spontaneous recovery oftener than the polycystic. The duration of life is greater, also, in the monocystic. The cases in which there is no great amount of solid matter, are more favorable for treatment, than those in which the solid is predominant, but ordinarily, the more of solid material, the less rapid the growth and, consequently, the longer the duration of life in the absence of interference. How long has the tumor been in attaining the present size? If several years have elapsed since the patient was aware of its presence, it will probably continue to increase slowly, unless, as is sometimes the case, more activity has lately been observed, and a tumor that had formerly grown very slowly, and required a number of years to acquire half its size, has grown the rest in a few months. In this last, there is every probability of a rapidly fatal course. Again, if the patient has not known any increase of size, until within a few months past, and yet is quite large, the prognosis is bad. Our prognosis is influenced by age, to a considerable extent; occurring in young persons, it is more likely to advance rapidly, than in old ones. A woman at forty, is not so apt to develop an ovarian dropsy so rapidly, as one at sixteen to twenty.

Ovarian dropsy will advance less rapidly after menstruation ceases, than before, and the earlier in menstrual life, the more rapidly it will advance. The prognosis, as a general thing, therefore, is worse in the young than the old. If we should decide the question, how long will she live by age, we should speak more favorably to the woman advanced in age. The fact, too, of the ordinary chances for the length of life being in favor of the young, she is more likely to fall a victim to the advance of the disease, than the older. We must take into consideration the circumstances that complicate it in the last stages, when we examine particular instances.

The inflammation, the pressure upon the rectum, bladder, stomach, bowels, and above all the kidneys, the nervous system, the muscular system, nutrition, as shown by the signs of emaciation or otherwise, should all be carefully scrutinized.

---

### IV.—Diagnosis of Ovarian Dropsy.

The diagnosis of ovarian tumors, when tolerably large, and not complicated with more than ordinarily embarrassing circumstances, is not difficult; but instances do occur where the matter is far otherwise, and a positive opinion cannot, with propriety, be given. For the most part, the diagnosis is sought to be perfected with a view to treatment, and we ought not *always* to be too solicitous about exactitude in this respect, until the urgency of the case demands it for the sake of treatment. When it is desirable to enquire particularly into the diagnosis, as a general thing we wish to know, 1st. Whether the case under examination *is* an ovarian tumor. 2d. What sort it is, solid, fluid, or mixed. 3d. If fluid, whether simple or multiple,—(multilocular or unilocular—polycystic or monocystic). 4th. Whether adherent or not. All these questions are very important, so far as our attempts to cure the case are concerned, and without an intelligent solution of them, our treatment will be experimental. Almost all our diagnostic means are physical; the history and rational symptoms, although valuable, are much less so than personal inspection with various physical appliances. The utmost freedom of examination, therefore, should be insisted upon, before our patient can, of right, demand our opinion. This will neither be asked nor given until the case has assumed something of gravity and importance. I have known medical men to give an opinion on imperfect examinations, that they were afterwards under the necessity of reversing; and I think, as a general thing, more errors of diagnosis in female diseases are committed by the imperfect use of our now highly improved measures of exploration, than originate in any other way.

The history will afford us in many cases, however, very valuable aid in arriving at correct conclusions. It is now pretty well determined that the average duration is about three years. In this time, it will spontaneously produce fatal effects, by great size and extreme distension, and the resulting damage. This is longer than pregnancy, and a shorter time than solid fibrous growths will ordinarily reach the same results. The age at which they are most likely to occur is an average of 26 years, according to BROWN,—although, they may occur at any time during the active condition of the sexual functions, while the ovaria are subject to menstrual congestions and their effects. Quite a large number of cases make their first appearance in early menstrual life. I knew one in which the beginning of the tumor must have been simultaneous with, if not antecedent to, the commencement of the function of menstruation. Fibrous growths of the uterus are not likely to begin so soon. Their increase after being first observed, is comparatively rapid, more so in the young, than those somewhat advanced in age. They are not usually attended with pain in their own proper substance; though, this is not always so, for the congestion and hyper-excitement may be attended with pain and soreness. The functional disturbance, in their early stages, occurs in the pelvic viscera:—1st. On account of pressure, such as tenesmus, dysuria, dragging or weight in the pelvis; and 2d, imperfect menstruation. Sometimes the menses are suppressed, scanty and painful, but often no deviation from the propriety of health is observed. The main thing in the history of the case, in this respect, is to remember that the symptoms point in the beginning to trouble in the pelvis. It is generally, or at least sometimes, stated that the tumor rises from one iliac region and continnes to occupy one side for some time. This, I think is the exception to the rule, that they are at first central (and by Dr. Frederick Bird is considered an evidence of adhesion.) They probably, when large enough to overcome the support of their peritoneal envelope fall into the *cul-de-sac* of Douglas behind the uterus; and then, as they grow, come up in front of the promontory of the sacrum, until they are large enough to be felt above the pubis, having their point of support in the hollow of the sacrum, instead of one of the iliac fossa. The patient will usually speak of it as a lump, instead of saying that she is swollen, as in pregnancy. The abdomen does not enlarge generally, according to her statements, but by a tumor rising in it. She has watched it coming up out of the pelvis, and not starting from above or from one side, and encroaching upon the abdomen from either of those directions. The knowledge derived by physical examination, as already stated, is most valuable; and while the modes of procedure are the same, and applicable to all stages of growth and enlargement of the tumor, we will be able better to describe and understand them, as made use of for one that has arisen from the pelvis and pretty thoroughly filled the abdominal cavity,—a tumor that has become obvious, and from which our patient is solicitous of being relieved. The means afforded us for physical examination are—1st, palpation, 2d, percussion, 3d, auscultation, 4th, vaginal and rectal digital examination, 5th, examination with the sound or uterine probe. These may be used separately, or combined in any given case, some being more valuable in some cases, and others in different ones. Exploring instruments, chemical tests, and the microscope may also be used to great advantage. Palpation is of very little use while the tumor is still in the pelvis, and can only be of avail in conjunction with the vaginal touch or the uterine probe; as it rises into the abdomen, however, this process of examination comes into use, independently. In this condition, we can examine the consistence, size, shape and mobility of the growth, and form some opinion as to its adhesion to the walls of the abdomen, and its primary attachments. In conjunction with some other processes of examination, it is more useful at this stage and size.

In the ordinary condition of the contents of the abdomen, the intestines lie in contact with the anterior and lateral walls, except in the right and left hypochrondiac, where the liver over a considerable space, and the spleen, a smaller, displace them. In consequence of this state of things, the resonance caused by the gas in the alimentary tube extends all over the anterior and lateral walls, save the above exceptions. Dulness upon percussion, therefore, indicates a change in this respect. The mesenteric attachments between the posterior wall of the abdomen and intestinal tube prevent them from being separated to any considerable extent, hence tumors occupying much space are apt to displace and get anterior to the latter. If the tumor springs from the pelvis, this is particularly likely to be the case, as well from the above facts as the direction given to it by the axis of the superior strait; thus it is with the gravid uterus, uterine fibrous growths and ovarian enlargements. Growths from the pelvis perhaps more completely gain the anterior position than any other sort, unless it be such as are attached to the anterior wall originally. It may be observed too, that it takes a larger growth to disengage itself from intestinal resonance, when arising from the posterior wall than from any other situation in that cavity.

By percussion we may make out the boundaries, positions, and, to some extent, attachment and contents of an abdominal tumor. We should begin at the pubis, and follow a line upward to the ensiform cartilage; by so doing we will ascertain the central perpendicular extent. A good plan is to make four or five perpendicular explorations of this kind each side of the median line, extending the whole length of the abdominal cavity. After this has been done, we may proceed, by right angles to these lines, to examine the abdomen crosswise, from its lower to its upper boundary. We will seldom miss any important growth by this mode of proceeding. If there is any doubt or obscurity, our pressure should be sufficient to bring out something of the flatness of sound from the spine, kidneys, etc. If we discover any point of sufficiently defined dullness to impress us with the idea of a tumor, we should, by percussing explorations, proceed from the point of greatest dullness to its circumference in every direction. In this way of examining, we will be able to trace it up the side to the hypochondriac regions, down into the pelvis, or define it so perfectly as to decide what must be its place of origin. Percussion and palpation will often enable us to determine the contents of a tumor as to its solidity, or fluidity. Placing the finger on one side of the tumor, while we percuss the other, if the contents are wholly fluid, a wave of liquid will be set in motion on the side struck, and traverse the space to the one of the opposite; if solid, of course nothing of this kind will take place, and the impulse will be given to the whole substance of the growth. Should the contents be fluid, separated by a number of partitions, the wave or fluctuation will be less distinct than in the one where no such division exists; but in fact the obscurity is so great, that we will be at a loss by this management to decide whether the contents are solid or fluid. A slight variation of this combination of tact and percussion will often clear it up however. When we wish to ascertain whether the fluid is contained in several cysts, we should place the pulp of the fingers of the left hand in the centre of the tumor, and then percuss with those of the right, first very near, then gradually increasing the distance between them, until we find a point at which the fluctuation becomes less distinct; this is the margin of the cyst over which our left fingers are placed. Still keeping them in position, we percuss around in every direction, until we have made out the boundary and size of the cyst under examination, when we may move the fixed fingers to its margin, and commence the same process around this point. Proceeding in this way from one point in the abdomen to another, in most instances we may trace the outline of all the cysts superficially situated, and thus enumerate them, and learn their relation and absolute size. If solid bodies of whatever structure are incorporated in the mass and superficially situated, they may be detected,—their relative position, size, etc., determined with some accuracy. After tapping, when the abdomen is lessened, its walls lax and soft, palpation and percussion, singly or combined, become more demonstrative than before this operation. It not unfrequently is necessary, on account of the sensitiveness of the patient, when the tumor is small and the abdominal muscles much under control of the will or reflex excitation, to administer chloroform until unconsciousness is induced, and the influence should often be so profound as to abolish reflex sensibility. In cases where there is inflammatory tenderness, the chloroform will give us a freedom of examination which is indispensable to an accurate diagnosis, and that we could not without it, by any possibility obtain. Palpation and percussion may both be practiced ordinarily as well with the patient in the recumbent position on the back, with knees drawn up, shoulders elevated, and the abdomen stripped quite bare of covering; in many instances, however, variation of posture is indispensable to definite results—the standing, prone, etc. Very little need be said in this place about auscultation, as it is only applicable to the diagnosis between it and pregnancy, and will be dwelt upon when I come to speak of that more particularly. Vaginal and rectal digital examinations were among the first physical means employed in the diseases of females; and in ovarian disease are proper, and should not be dispensed with. The pelvis should be carefully surveyed by this method. The attachments, consistence and relations of the diseased mass to the various organs in this cavity should be carefully noted. The uterus, rectum and bladder so far as practicable, ought to be examined with reference to their healthy condition, position and involvement. Combined with external palpation, we may examine the tumor more thoroughly than with either one alone. Two fingers introduced into the vagina after the disease has somewhat advanced, and pressed firmly upward against it will perceive any impulse imparted to the tumor above. With the left hand, if we press downward toward the pelvis, we may feel the motion of the diseased accumulation downward, and if the sudden impulse of percussion is applied above, we may feel an impression from its contents; if fluid, a wave or sense of fluctuation, if solid, the deadened impulse always given in such cases. When the tumor is small, and occupies the posterior peritoneal *cul-de-sac*, by introducing one finger in the rectum, and the other into the vagina, the tumor may be included between them, and thus examined with more accuracy than with either alone.

Dr. Simpson has taught us how to extend our examinations into the uterus, so that our information in this direction is very materially increased by the use of the probe, mounted upon a handle. Members of the profession, who appreciate the labors of Dr. Simpson, have by consent, named the instrument, the improvements and uses of which he has so ably promulgated, *Simpson's sound.* The most useful form, I think, of the sound, is round and plain, without any notches or edges, with the button end about two lines in diameter. Back of the head or button end, the wire should be about half the thickness of the end; from this, the wire should gradually increase in size towards the handle, until it is near a quarter of an inch thick. The material of which it is made should be soft enough to be bent into any shape we choose, and yet sufficiently firm to retain the flexure thus given.

The sound may be introduced into the uterus, and varied in its direction while we gently urge it forward to the extremity of the uterine cavity. The only obstacle a sound of the dimensions I have mentioned will meet with, will arise from want of correspondence in direction with the direction of the cavity in a uterus of ordinary size. The most simple and ready revelation of the sound or probe, is the direction and length of the uterine cavity. From this knowledge, much valuable deduction may be drawn. But it is employed for determining the relation of the uterus to pelvic tumors, according to the ingenious directions of Dr. Simpson, very handily and to excellent pupose. While the sound is in the cavity of the uterus, this organ may be fixed by holding the instrument firmly in one position, or be moved in any direction, if not restrained by adhesion or accretional attachment to the diseased mass, or to some other organ. If the uterus be fixed, and the tumor moved by its side, or from it with the fingers introduced for the purpose, the motion will be felt affecting the uterus, through the attachments. On the other hand, if we watch the motion of the tumor with the fingers while the uterus is moved, the attachment or not will be determined, or the uterus may be moved in one direction, and the tumor in another. In this way, their attachments may be pretty certainly diagnosticated. The sound may be employed in the uterus with one hand, while palpation on the abdominal surface is effected with the other; and if the uterus reaches above the pubis, the distance the probe is separated from the external hand, or its relation with the median line of the abdomen, or the main bulk of the growth, will enable us to determine some interesting problems. The motion received by the sound from the pressure of the hand without, or *vice versa*, is of important significance, as will be more apparent as we advance. When, however, from all these sources of inquiry we fail to get a sufficiently definite answer, there is still another physical means of diagnosis which we are justified in employing, viz: *exploration.* If by means of an exploring needle or trochar we draw off a small quantity of fluid, it may be subjected to microscopic and chemical tests that will often enable us to determine the nature of the disease. Dr. G. HUGHES BENNETT, in a paper on ovarian disease in *Edinburg Med. and Surg. Journal*, quoted by BROWN says, as the result of his microscopic "examinations of different specimens of ovarian fluid, that the most constant characteristic of such fluid is its containing in greater, or less abundance, cells gorged with granules; and in addition circumambient granules having the same measurement as those encompassed by the cell-wall. At one time, I considered the size of these granules (if they can properly be so called) was constant; but subsequent observations have convinced me of the incorrectness of this conclusion—the size of the gorged cells and granules varies greatly, even in the fluids from different cysts of the same ovary." There can be no question but that the nature of the fluid contained in these cysts is, in all its essential features, pretty constantly the same in the early stages of progress; but it is equally true that as they grow large enough to be influenced by pressure or other external causes, their composition, microscopically, must vary. The chemical nature of this fluid is more constant. It is alkaline in reaction, and highly albuminous; always coagulating when boiled or submitted to the action of strong acids. Another sort of exploratory examination is the evacuation of a part or the whole of the fluid, provided fluid flows through the tube. If so much fluid is drawn off as to greatly relax the abdominal parietes, the contents of this cavity may be much more thoroughly examined by palpation and percussion, and the nature of the growth ascertained quite definitely. We can then handle the abdominal contents, so as to trace any tumor within it to its attachments, get its precise size and shape, consistence, etc. Or if there should be no morbid growth, it will become quite evident, after a greater or less evacuation of the fluid by tapping.

After having passed in review, as above, the items of general diagnosis of ovarian tumors, I propose to enter upon a differential view of the subject; for there are conditions of disease and health of the contents of the female pelvis and abdomen, which they may be mistaken for. The following list is given by Mr. J. Baker Brown, of conditions that may be mistaken for ovarian tumor. "1st. Retroversion and retroflexion. 2d. Tumors of the uterus,—solid, fibrous, or fibro-cystic. 3d. Pregnancy. 4th. Pregnancy complicating ovarian dropsy. 5th. Cystic tumors of abdomen. 6th. Distended bladder. 7th. Accumulation of gas in the intestines. 8th. Accumulation of fœces in the intestines. 9th. Enlargement of the liver, spleen or kidneys, or tumors connected with the viscera. 10th. Recto-vaginal hernia, and displacement of the ovary. 11th. Pelvic abscess. 12th. Retention of menstrual fluid from imperforate hymen or closure of the os uteri. 13th. Hydrometra."

In cases of retroversion, or retroflexion, if minute examination with the finger per vaginum and rectum fail, and the symptoms are of a character to make a correct diagnosis important, the uterine probe will at once determine the distinction. In some instances, we might be quite unable to distinguish a small ovarian tumor from an impregnated retroverted uterus. Our proper plan in such cases is to await the peremptory demand for the knowledge, and then take the risk of introducing the probe; remembering the position of the mouth of the womb in retroversion, that it is not only near the pubis, but directed upwards as well as forwards, and that the os, in cases of misplacement by the tumor, is not directed upward, but nearly always downward,—certainly never, so far as my experience and reading goes, above the horizontal position. The probe will be equally available in examining the retroflected organ, and I think the probe should always be used where pregnancy is not strongly suspected. Should we feel much doubt of the existence of pregnancy in connection with retroversion, it would be better to lift the tumor out of the pelvis, when if it were retroversion, the uterus would be restored to its natural position, with the os near the centre of the pelvis. In endeavoring to distinguish between ovarian and uterine tumors, we should bear in mind that the latter almost invariably change the length and size of the cavity of the uterus. Where the sound is used, it will pass further than if the uterus was not involved. The rationale of this increase of size of the uterus, so generally found to be present, is, I presume, connected with the fact that the development of a tumor in, or from the walls of that organ induces general hypertrophy to some extent, as these growths are found to be a hypertrophy of some one of the uterine tissues. The tissues generally involved are the fibrous or mucous, as in hard or soft polypi from the internal, or hard from the external walls or intramural fibrous tumors. Uterine tumors are so intimately connected with the uterus, that this organ cannot be moved without imparting more or less motion to the tumor, nor can the tumor on the other hand be moved, without in a similar way affecting that organ. This is not the case with ovarian tumors; they are so loosely connected with the womb that considerable motion is allowable without the other partaking of it. In the sound, we have the means of moving or fixing the uterus, and with the finger may watch the effect of motion upon the one or the other, as the case may be. When a fibro-cystic tumor is developed upon the uterus, containing fluid, the examination to ascertain whether there is an attachment with the uterus, and with a view to learn the length of the cavity, will give us clear notions of the matter. When we are satisfied that pregnancy cannot be the condition we may explore or tap it, as an additional means of accuracy.

Hard or fluid tumors arising from a distant organ or part of the abdomen, would have a different history from the ovarian tumor. If our patient is intelligent, her observation as to the place where first noticed, should be relied upon as valuable knowledge respecting the probable point of origin. Ascites, when excessive, may sometimes be mistaken for ovarian tumor, but the latter is more frequently taken for the former. When the patient lies on her back with the knees drawn up, so as much as possible to relax the muscles, and the abdomen is entirely exposed, in ascites the tumidity will be rotund, filling out in every direction, and will particularly bulge the depending portions. The flanks will both be full, the abdominal protrusion commence at the edges of the ribs, and will be equally soft at every point; fluctuation will be greatest at the most dependant parts, and resonance entirely absent; fluctuation will scarcely be perceptible in the highest part of the abdomen. These circumstances will remain the same under any change of position. If the patient stand up, the dulness is in the hypogastric and iliac regions; if she lie on her side, the dullness and fluctuation on the lower side, resonance and absence on the upper side. All this results from the water freely settling into the lowest points, let them be what they may. In ovarian tumor, alteration of position, from erect to recumbent, or from supine to prone makes no difference in the places where resonance and fluctuation are found. They are manifested always in the same places. When the patient lies on the back, the flanks are resonant, the unbilical region dull; fluctuation is not observed in the flank in any position; it is apt to be greatest under any posture in the middle of the abdomen. When the abdomen is exposed for inspection, there is marked irregularity in its rotundity; and I think, ordinarily the flanks, one or both, are flat; one side is apt to bulge more than the other. Probably, there is more than one rather prominent *region*—it may be several—there is more hardness and tension, not the flabby swaying under slight influences, so common in ascites. An important class of circumstances is the pathological conditions almost always present in ascites; it seldom occurs in persons in the enjoyment of good health in every other respect. There is organic disease of the kidneys, liver, spleen, heart, lungs, or subacute peritonitis. Or there may be some cachexia from miasma, poison, the poison,—or other bad influence of particular places of residence, occupation, habits, or time of life, etc. There is some notable and grave pathological accompaniment of abdominal dropsy, which preceded the swelling; whereas the ill health in ovarian dropsy is the effect, and not the cause. We generally find that women preserve a good condition of health in ovarian disease, until far advanced; and disordered functions come almost always as the result of great pressure upon the suffering organ. A complication of ascites with ovarian dropsy obscures our diagnosis very much. If the ascites is great, and the ovarian disease not so considerable, the tumor will be felt floating about as it were, when the patient changes position, in the abundant fluid. Excluding by our diagnostic examination, every other disease, and leaving the question between them alone, we are justified in exploration, and even tapping. By the former, we come in possession of a specimen fluid, which, when submitted to chemical and microscopical investigations, is almost conclusive; by the latter we partially empty the abdominal cavity, and relax the walls, so that we can examine its contents with great freedom. If the fluid is ovarian, it will be highly albuminous, and possess the microscopical qualities I have before mentioned; if it be ascitic, the properties are those of serum found exuded anywhere from pressure or inflammation. There will be very little, if any, albumen, no epithelial cell, and the corpuscles described by Dr. BENNETT.

It will occur very seldom that the question between pregnancy and ovarian disease will become so urgent, that it may not safely be left to time. I can conceive no time or circumstances under which great doubt as to which of these two conditions were present, but in the early stages of either, while in the pelvic cavity; and unless great pressure on the organs contained in it make delay hazardous, we should not interfere, but content ourselves to wait until the obvious evidences, as quickening and motions of the child, declare the existence of pregnancy, or until so much time had elapsed without any such signs as to throw great doubt upon the subject. At such times the tumor is high above the pelvis, and may be subjected to any searching examination we may choose. Auscultation then becomes valuable and perfectly reliable, when properly practiced, in determining the presence of normal pregnancy.

Frequent examinations with the stethoscope or ear, in various positions, should be patiently and perseveringly practiced before we should be satisfied to risk means of a hazardous nature, that will enable us positively to decide the question. After having repeatedly thus explored the abdomen, without any sign of a live fœtus, we may use the probe to examine the whereabouts and size of the uterus. No mistake will survive the test of this instrument. If I were not to explain myself a little more upon this point, I might incur the charge of rashness for recommending the sound where any doubts exist. It would be rash to use the sound until all the differential signs of pregnancy had failed, and even then, unless the urgent demand caused by the influence upon the health forbids us to wait longer for a decision. It is only in extreme cases, where the symptoms and signs derived from the breasts, menstruation, nausea, pigmentary deposits, and auscultation, had all failed, and yet I was obliged to act at once for the safety of the patient, that I should consent to use the sound. Then I would use it as the more innocent of the demonstrative tests, and as a *dernier ressort.* It is certainly more innocent than the exploring needle or evacuating trochar and equally demonstrative. The worst effect its careful use could have, would be to produce abortion or premature birth, either of which would be more likely to remove the urgency of the symptoms, than do harm. I have recently seen an instance of obscurity of diagnosis, from the existence of a pregnancy of eight and a half months duration, decided by the probe, which caused the discharge of a mummified fœtus of less than four months growth, and as a matter of course, almost cured the patient.

Pregnancy complicated with ovarian dropsy, may be very perplexing to diagnosticate. At a sufficiently advanced stage of pregnancy, auscultation will reveal the fact, and it cannot be a matter of very great importance to decide the nature of the tumor until the termination of the process of gestation, as a general thing, and if it should, we may proceed in its examination according to the plans heretofore indicated. This complication may continue up to the time of labor, and embarrass that process. Such embarrassment will not arise, if the tumor is large enough to rise out of the pelvis, but if it is still within that cavity, and gets below the child's head it may arrest its progress. There are very few collections or growths that can be, in such conditions, mistaken for this. In pelvic abscess, there will be inflammatory tenderness and heat. The most likely of all others, is a prolapsed bladder. Our diagnosis, however, will be easily effected by using the catheter, when, if it is the bladder, emptying causes its collapse and the entire disappearance of the tumor. But if, after the complete evacuation of the bladder, there is yet a tumor containing fluid, exploration should be resorted to. This will clear up the diagnosis, provided the exploring trochar is large enough to evacuate a part or the whole of its contents. There are other fluid tumors, arising from the broad ligaments near the ovary, probably dependent upon a great increase of one or more of those transparent cells of serum, so generally seen by looking through this peritoneal duplicature, towards the light. These may be mistaken for actual ovarian cysts, (and these are doubtless the cases of ovarian disease that are permanently cured by a single tapping.) I think no means of diagnosis now known, would enable us to decide, with any certainty, between the two. Exploration and chemical and microscopic examination of the fluid would throw some light upon, but not necessarilly clear up the case. Cystic tumors of the abdomen, arising from other points, and hydatids of the peritoneal cavity, can be distinguished with certainty in no way except by exploration and examination of the contents. The history will, if carefully and intelligently detailed, show something, perhaps, that we may seize upon to aid us. The case should commence, if ovarian, in a tumor arising from the pelvis gradually *ascending* into the abdomen. If abdominal, it is first noticed in that cavity, and may descend until it occupies all the abdomen, and then the pelvis also. If hydatid, the increase is mere tumidity—not a well-defined tumor, and it commences in the abdomen.

The distended bladder, accumulation of gas in the intestines, or of fœces, ought not, in the present state of our science, to embarrass us any longer than the catheter or a cathartic could be brought to bear upon the case. As soon as the bladder is emptied it will collapse. The gas in the bowels causes tympanitis of the abdomen, and thus ought to be detected. The accumulation of fœces can be removed, when the tumor will be gone. Hysterical distension of the abdomen, said to simulate pregnancy, ovarian, uterine, and other tumors, entirely disappears under the influence of chloroform, as shown by Prof. SIMPSON, on many occasions.

Visceral enlargement, as liver, spleen, kidneys, and tumors growing from them, are not unfrequently mistaken for these tumors. I have a patient now laboring under enlargement of the spleen, who has been told more than once, that she had ovarian disease. Unless the enlargement of the liver or spleen is excessive, I cannot see how a mistake can be possible. The history as to where the tumor was first observed should be carefully traced. If either of these it has descended. I have not seen a liver or spleen occupying the cavity of the abdomen so completely, but that its well-defined edge could be felt for a considerable distance, and this edge is always below, while the upper boundary is less defined or traceable beneath the ribs. I have, on several occasions, seen the spleen enlarged and dislocated, occupying the left iliac region, and reaching up toward the hypochondriac, but there are always sharp edges somewhere. This is not the case in ovarian dropsy; it is round, somewhat even, and elastic to the touch.

Mr. BROWN mentions recto-vaginal hernia and dislocation of the ovary into the *cul de sac* of Douglass. The diagnosis would be difficult, and as unimportant, unless in exceptional cases. The great importance of a correct diagnosis is based upon the urgent symptoms and tendency of disease. If the tumor is so small, and situated in the *cul de sac*, we can not only afford to wait for further developments, but it is our duty to do so.

Retention of menstrual fluid, from imperforate hymen, (or other obstruction to its outlet,)—hydrometra,—as soon as we have by physical examination, history, the rational symptoms, and decided that the patient is not pregnant, the finger and sound will clear up all doubts in a short time. Obstructions will be ascertained or overcome by them, and our misgiving dispelled.

Supposing our diagnosis complete, as to its being an ovarian tumor, we have yet to learn, for the more intelligent treatment, several other things; among these are,—What are the the contents and construction of it? Is it monocystic or polycystic? Are its contents partly solid, or wholly fluid? Although, probably, not always possible to decide these questions without exploratory operations, we have some means of clearing them up. A diligent and careful examination by percussion and inspection will enable us to judge correctly, in most cases, whether the tumor is monocystic or polycystic, or otherwise. If monocystic, the tumor is regular in its rotundity, and outline, if polycystic, there is something of elevation, made out best by sliding the hand over the surface. Fluctuation, caused by percussion, is the same in all directions and from all points of it. In polycystic, it is very obscure, except over partial measurements. The fingers placed near each other over the same cyst feel the fluctuation very sensibly; but when one is removed so as to pass over the partition between it and the next cyst, the fluctuation becomes more obscure. By examining all parts with both hands, separating and approximating each other, we may make out the dimensions and situation of the cyst, which lies in contact with the abdominal walls. The fluctuation, or its absence, will determine whether a given part of the tumor is solid or fluid. The hard parts of an ovarian tumor, are, almost invariably, at the bottom of the tumor, and may be reached by the finger per vaginum. While our fingers are in contact with the base of the tumor in the pelvis, if it is wholly fluid, we may feel fluctuation, if the top of the tumor is struck with the other hand. If a solid part intervenes between our two hands, fluctuation would not be experienced.

A very important and difficult point in our diagnosis, is the presence or absence of adhesions to the viscera or walls of the abdomen. The nature of our practice will depend very much upon the determination of this question. Adhesions are very much more frequent in front than behind the tumor; to the walls of the abdomen than to the viscera. There is no way, so far as I am informed, to decide whether there exist adhesions to the moveable viscera, and unless when the tumor is small and moveable, whether it is adherent behind or not. If there is none in front, there is not likely to be any behind. If there has been no peritoneal inflammation nor signs of other inflammation in the abdomen, we should be encouraged to hope that there is no adhesion, for there can be little doubt that adhesions are more frequently the result of organized inflammatory effusions between the two surfaces of the peritoneum, than all other causes. This is the explanation of adhesions, following punctures with the trochar or exploring needle. If the patient has always enjoyed good health, and not suffered from pains in the abdomen, there has *probably* been no inflammation, and therefore no adhesion. It should not be inferred, however, that there is *certainly* no adhesion, because there has not been any inflammatory symptom; for either these symptoms may have been so obscure as to have escaped observation, or the adhesion may have resulted from some other cause, in many instances. Certain it is, adhesions do exist without anything in the history of the case leading us to suspect them. We should enquire whether any operation, pressure or other curative measures of a local nature have been used, as almost all of them endanger adhesions to the front wall of the abdomen.

If there is not too great tension, we may gather the walls of the abdomen up and move them over the tumor to a sufficient extent to establish their freedom from adhesions. In fact, there is not much adhesion where we can thus raise the muscles in folds. Another way is to place one hand on each side of the abdomen, with the palms applied flatly to its surface, and, pressing with some firmness against the tumor, we may attempt to slide the walls over the growth. As adhesions are not apt to be universal over the anterior part, a difference in the mobility of certain parts would lead us to point out the probable localities and extent of these complications. The hands should be moved from place to place until all the surface has been thus examined. This, when properly and carefully done, is a valuable means of examination. When the patient is lying on her back, with the walls as much relaxed as possible, if she make several deep inspirations we may observe the muscles moving over the growth, or see that the whole mass is carried down together. When in the same position, if the patient will make an effort by the abdominal muscles alone to elevate the chest, or throw her head forward, if there is no adhesion under the rectus muscles, these organs will show their bellies distinctly, by swelling up in their middle portion. If there is adhesion on one side and not on the other, the muscle that is free will bulge, while the other remains flat and undefined in any such way. If both are adherent, the whole will remain smooth under the efforts. If the adhesion is at the lower part, and not at the upper, the bulging will take place above, and not at all below. Now, by carefully watching the effect of this sort of effort, we may discover adhesions with some degree of accuracy, and somewhat define their extent.

